# Rebound of multiple infections and prevalence of anti-malarial resistance associated markers following malaria upsurges in Dielmo village, Senegal, West Africa

**DOI:** 10.1186/s12936-023-04694-0

**Published:** 2023-09-05

**Authors:** Amélé Nyedzie Wotodjo, Mary Aigbiremo Oboh, Souleymane Doucoure, Nafissatou Diagne, Fatoumata Diène-Sarr, Makhtar Niang, Jean-François Trape, Cheikh Sokhna, Alfred Amambua-Ngwa, Umberto D’Alessandro

**Affiliations:** 1 VITROME, UMR 257 IRD, Campus UCAD-IRD, Dakar, Senegal; 2 Medical Research Council Unit, London School of Hygiene and Tropical Medicine, Fajara, The Gambia; 3 Department of Biological Sciences, University of Medical Sciences, Ondo, Nigeria; 4 Department of Biomedical Sciences, Rochester Institute of Technology, Rochester, NY USA; 5 Institut Pasteur de Dakar, 36 Avenue Pasteur, 220 Dakar, Senegal; 6 UMR MIVEGEC, Laboratoire de Paludologie et Zoologie Médicale, IRD, Dakar, Senegal

**Keywords:** Anti-malarial drugs, Resistance, *Pfcrt*, *Pfmdr1* 86, *Pfmdr1* 184, Malaria upsurges, Dielmo, Senegal

## Abstract

**Background:**

Thanks to the scale up of malaria control interventions, the malaria burden in Senegal has decreased substantially to the point that the National Malaria Control Programme plans to achieve malaria elimination by 2030. To guide such efforts, measuring and monitoring parasite population evolution and anti-malarial drugs resistance is extremely important. Information on the prevalence of parasite mutations related to drug resistance can provide a first signal of emergence, introduction and selection that can help with refining drug interventions. The aim of this study was to analyse the prevalence of anti-malarial drug resistance-associated markers before and after the implementation of artemisinin-based combination therapy (ACT) from 2005 to 2014 in Dielmo, a model site for malaria intervention studies in Senegal.

**Methods:**

Samples from both malaria patients and *Plasmodium falciparum* asymptomatic carriers were analysed with high resolution melting (HRM) technique to genotype *P. falciparum* chloroquine resistance transporter (*Pfcrt*) gene haplotypes and multidrug-resistant protein 1 (*Pfmdr1*) gene at codons N86 and Y184.

**Results:**

Among the 539 samples analysed, 474, 486, and 511 were successfully genotyped for *Pfmdr1* N86, Y184, and *Pfcrt*, respectively. The prevalence of drug resistance markers was high, particularly during the malaria upsurges. Following the scale-up in bed net distribution, only the mutant (86F-like) variant of *Pfmdr1* 86 was present while during the malaria upsurges the predominance of two types 86Y-86N (43%) and 86F-like (56%) were observed. Most infections (87%) carried the wild type Y-allele at *Pfmdr1* 184 during the period of nets scale-up while during the malaria upsurges only 16% of infections had wild type and 79% of infections had mixed (mutant/wild) type. The frequency of the mixed genotypes SVMNT-like_CVMNK and SVMNT-like_CVIET within *Pfcrt* gene was particularly low during bednet scale up. Their frequency increased significantly (P < 0.001) during the malaria upsurges.

**Conclusion:**

This data demonstrated the effect of multiple interventions on the dynamics of drug resistance-associated mutations in the main malaria parasite *P. falciparum* in an endemic village in Senegal. Monitoring drug resistance markers should be conducted periodically to detect threats of emergence or resurgence that could compromise the efficacy of anti-malarial drugs.

**Supplementary Information:**

The online version contains supplementary material available at 10.1186/s12936-023-04694-0.

## Background

About 20 years ago, the recommended treatment for uncomplicated malaria changed from monotherapy with either chloroquine (CQ) or sulfadoxine-pyrimethamine (SP), to artemisinin-based combination therapy (ACT) [1]. Such a change, besides providing a highly efficacious treatment to malaria patients, aimed at preventing or at least delaying the emergence and spread of drug resistance [2]. In Senegal, following the implementation of ACT, using artesunate-amodiaquine (ASAQ) and artemether-lumefantrine (AL) as first-line drug combinations in 2006 [3, 4], and the large scale implementation of insecticide-treated nets (ITNs) in 2008, malaria morbidity and mortality decreased from 22.2% and 18.2% in 2007 to 5.6% and 7.1% in 2008, respectively [5, 6].

The National Malaria Control Programme (NMCP) currently aims to achieve malaria elimination by 2030. However, this may be threatened by the emergence and spread of resistance to artemisinin and/or its partner drugs. Indeed, the spread of chloroquine resistance in the 90 s was associated with 2- or threefold increase in malaria deaths and admissions from severe malaria [7]. Artemisinin resistance, first reported in Asia [8, 9], has been recently detected in Rwanda and Uganda [10, 11]. Therefore, monitoring anti-malarial drug resistance is extremely important to guide elimination efforts.

Molecular markers can be used to detect and monitor the early emergence and subsequent spread of drug resistance [12, 13]. Mutations on the *Plasmodium falciparum* multidrug-resistant protein 1 (*Pfmdr1*) gene are associated with reduced amodiaquine (AQ) and artesunate (AS) sensitivity. *Pfmdr1* single nucleotide polymorphisms (SNPs) at codons N86 and Y184 have been correlated with AL or ASAQ drug pressure [14]. AQ resistance has been associated with mutation at codon N86 of the *Pfmdr1* gene, and cross-resistance between AQ and CQ has been observed [12, 15, 16]. CQ resistance has been associated with mutations at codons K76 of the *P. falciparum* chloroquine resistance transporter (*Pfcrt*) gene and at codon N86 of the *Pfmdr1* gene [16, 17]. Mutant haplotypes of *Pfcrt* gene are associated with CQ, AQ, and lumefantrine (LUM) resistance. These include specifically, the *Pfcrt* 72–76 codon representing either the CVIET or SVMNT haplotypes, depending on the geographical location (CVIET being common in Africa, and SVMNT common in South-east Asia) [18]. The SVMNT haplotype is required for AQ resistance [14]. In Senegal, *Pfcrt* and *Pfmdr1* mutations have been associated to decreased AQ, CQ and artemisinin sensitivity [19, 20]. Nevertheless, no artemisinin resistance mutation in the *pfkelch13* propeller domain (K13) has been reported in the country, despite sustained use of ACT [21, 22].

Although the malaria burden has decreased in Dielmo, Central Senegal, following the scale-up of ACT and ITNs [23], there have been two malaria upsurges over the last decade; the first between 2010 and 2011 and the second between 2013 and 2014 [24, 25]. These upsurges were associated with vector’s resistance to pyrethroid and low use of LLINs [24, 26]. However, the contribution of drug resistance in the malaria upsurges was not investigated. Hence, this study evaluated the prevalence of drug resistance molecular markers before and after the implementation of ACT in Dielmo, and during the two malaria upsurges.

## Methods

### Description of the study area and interventions

The study samples were recruited between 2005 and 2014 in Dielmo, a village in Central Senegal at 280 km south-east of Dakar, where a malaria research project has been ongoing since June 1990 [27]. Malaria in Dielmo was holoendemic, with perennial transmission, until 2009, when it became hypo-endemic with seasonal transmission [23]. Between 2005 and 2006, the first-line treatment for uncomplicated malaria was a combination of AQ and SP. This was replaced by ASAQ and AL until the end of the study period in 2014. Universal coverage with ITNs has been implemented in Dielmo since 2008, with distribution campaigns in 2011 and 2014. Further distribution campaigns were carried out in 2016 and 2019, outside the study period. Two malaria upsurges occurred in 2010–2011 and in 2013–2014 [24, 28] in Dielmo.

### Samples collection

Two types of samples were used for this study: the capillary blood samples and thick blood smears (TBS) from asymptomatic carriers and only TBS from symptomatic patients. TBS were prepared for microscopy; capillary blood samples were collected quarterly in microtubes from cross-sectional surveys carried out between 2005 and 2014. Therefore, for symptomatic patients, DNA extraction was performed only on TBS while for asymptomatic carriers both capillary blood and TBS (when blood samples were not available) were used for DNA extraction.

### DNA extraction, selected whole genome amplification and DNA quantification

DNA was extracted using Qiagen minikit, mostly following the manufacturer’s recommendations or with slight modifications. For TBS, 200 µl of PBS was used to resuspend the blood smear and DNA was eluted in 20 µl of elution Buffer. For capillary blood, DNA was eluted in 200 µl of elution Buffer.

Real time PCR (RT-PCR) targeting the *var* gene multi-copy acidic terminal sequence (varATS) was performed on the extracted DNA to confirm *P. falciparum* positivity as previously described [29]. Cycle threshold of < 39 was employed as discriminatory criteria for *P. falciparum* positive samples to be processed for other assays. *Plasmodium falciparum* selective whole genome amplification (Pf-sWGA) was performed on DNA extracted from TBS to increase the DNA concentration. The Pf-sWGA was performed using a volume of 9.5 µl of nuclease free water, 2.5 µl of Phi 29 Buffer, 15 unit (1.5 µl) of phi29 polymerase, 0.25 µl of BSA, 1 mM (2.5 µl) of dNTPs, 2.5 µM of primer each for a total of 10 primers and > 1.33 ng/µl of DNA in a total reaction volume of 25 µl.

### SNPs analysis with high resolution melting (HRM) method

Pf-sWGA DNA were normalised to 5 ng/µl for the high resolution melting (HRM) assay. The assay was performed using the 96 Light-Cycler Roche™ thermocycler [30]. Briefly, 0.7 µl of 10X primers (forward and reverse) were added to 5 µl of HRM Master Mix (Type-it HRM PCR kit (2000) – Qiagen Cat no: 206546) and 3.3 µl of nuclease-free water. One microlitre (1 µl) of DNA was added to the mix for a total reaction volume of 10 µl. The PCR cycling conditions were 95 °C during 300 s for preincubation followed by 50 cycles with denaturation step at 95 °C for 10 s, annealing at 55 °C for 30 s and elongation at 72 °C for 20 s. PCR amplification was immediately followed by data acquisition at melt temperature 40 °C for 1 s followed by 95 °C for 30 s and then 40 °C for 1 s. Analysis of acquired data was performed using the Light-cycler software after adjusting the sliding window to the appropriate melt curve for each target. The different types of polymorphisms were defined using Daniel’s nomenclature [30]. Wild and mutant alleles, and the negative control were added to each plate and used to call the alleles of each sample. Reference isolates from the malaria laboratory at the MRC Unit The Gambia were used as controls (Additional file 1: Table S1). The molecular markers targeted in this study are *Pfcrt* haplotype and *Pfmdr1* (N86, Y184). The sequences of the primers are given in Additional file 2: Table S2.

### Statistical analysis

Samples were classified by time of collection into four groups, namely before ACT implementation (year 2005); at the time of ACT implementation (year 2007); after ACT implementation and scale up of ITNs (years 2008–2009); and during the two malaria upsurges in 2010–2011 and 2013–2014. Chi-2 test was used to evaluate differences in prevalence of various alleles by period. The significance level was fixed at p = 0.05. Analyses were performed using Stata Software, version 11.0 (College Station, Texas, USA).

## Results

### Study population

Five hundred and thirty-nine samples (55.3%) out of 974 were positive by RT-PCR, of which 71.4% (385/539) were from patients with clinical malaria, and 28.6% (154/539) from asymptomatic cases; and 33% (180/539) were collected during the two malaria upsurges. The distribution of positive samples is shown in Table 1. There was a decline in parasite positive samples following scale up of interventions in 2008–2009. More than 40% (44%, 67/154) of positive samples among asymptomatic *P. falciparum* carriers were collected in 2007, when ACT became the first-line treatment.

**Table 1:** Number of *P. falciparum* positive samples by *var*ATS RT-PCR according to periods

	Asymptomatic	Symptomatic	Total
2005 (before ACT)	43 (28%)	73 (19%)	116 (22%)
2007 (ACT implementation)	67 (44%)	90 (23%)	157 (29%)
2008–2009 (ITNs scale up)	16 (10%)	70 (18%)	86 (16%)
2010–2011 and 2013–2014 (malaria upsurges period)	28 (18%)	152 (39%)	180 (33%)
Total	154	385	539

### *Pfmdr1* gene polymorphism at codon N86

**Fig.1:**
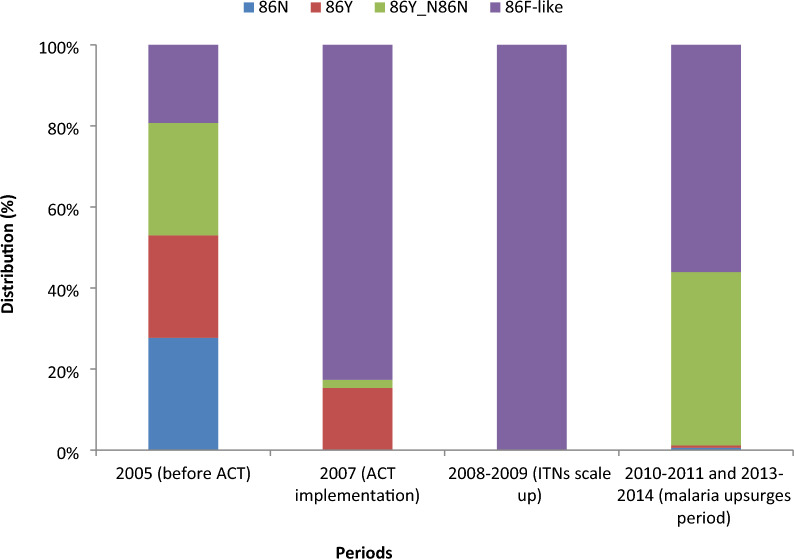
Distribution of *Pfmdr1* N86 haplotype in Dielmo *Plasmodium falciparum* isolates by period (474 genotypes)

Most samples (87.9%, 474/539) were successfully genotyped for *Pfmdr1* N86. In 2005, three types of alleles (wild-86N, mutant-86Y, mixed mutant/wild-86Y-86N), and 86F-like (this mutation is uncommon in natural parasite isolates in West Africa, so it is called 86F-like in this study by precaution) were present at similar prevalence, with wild type infections found mainly during this period (Fig. 1). Since 2007, most infections carried the 86F-like allele both in asymptomatic carriers and clinical cases, with an increase of the mixed mutant/wild type (86Y-86N) during the malaria upsurges (Table 2). In 2007, no mutant type was detected among clinical cases while 35% of asymptomatic carriers had mutant 86Y. In 2008 and 2009, the 86F-like type was the only allele present.

**Table 2:** Prevalence (%) of the *Pfmdr1* N86 haplotype by period in asymptomatic carriers and clinical cases

	N86	86Y	86Y-N86	86F-like
Asymptomatic carriers				
2005 (before ACT) (N = 51)	19 (37)	5 (10)	11 (22)	16 (31)
2007 (ACT implementation) (N = 66)	0 (0)	23 (35)	0 (0)	43 (65)
2008–2009 (ITNs scale up) (N = 11)	0 (0)	0 (0)	0 (0)	11 (100)
2010–2011 and 2013–2014 (malaria upsurges) (N = 28)	1 (4)	1 (4)	4 (14)	22 (79)
Symptomatic (clinical cases)				
2005 (before ACT) (N = 32)	4 (13)	16 (50)	12 (38)	0 (0)
2007 (ACT implementation) (N = 84)	0 (0)	0(0)	3 (4)	81 (96)
2008–2009 (ITNs scale up) (N = 57)	0 (0)	0 (0)	0 (0)	57 (100)
2010–2011 and 2013–2014 (malaria upsurges) (N = 145)	0 (0)	0 (0)	70 (48)	75 (52)
All				
2005 (before ACT) (N = 83)	23 (28)	21 (25)	23 (28)	16 (19)
2007 (ACT implementation) (N = 150)	0 (0)	23 (15)	3 (2)	124 (83)
2008–2009 (ITNs scale up) (N = 68)	0 (0)	0 (0)	0 (0)	68 (100)
2010–2011 and 2013–2014 (malaria upsurges) (N = 173)	1 (1)	1 (1)	74 (43)	97 (56)

### *Pfmdr1* gene polymorphism at codon Y184

**Fig. 2:**
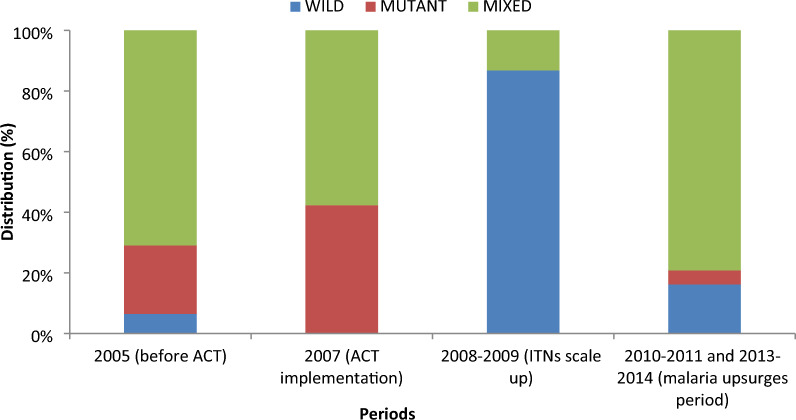
Distribution of *Pfmdr1* Y184 haplotype in Dielmo *Plasmodium falciparum* isolates by period (486 genotypes)

Almost all samples (90.2%, 486/539) were successfully genotyped for *Pfmdr1* Y184. The frequency of wild type parasites was extremely low throughout the study period, except for the years 2008–2009 when most infections carried the wild type, while mixed allele infections (mutant and wild) were predominant during the period of malaria upsurges (Fig. 2). Among asymptomatic carriers, mixed infections were more frequent in 2005 and at the time of malaria upsurges, in 2010–2011 and 2013–2014, whereas the mutant type was predominant during ACT implementation, in 2007 (Table 3).

**Table 3:** The number of infections and prevalence (%) of *Pfmdr1* 184 alleles (wild = Y, mutant = F or mixed = Y/F) by period in asymptomatic carriers and clinical cases

	Wild	Mutant	Mixed
Asymptomatic carriers			
2005 (before ACT) (N = 42)	1 (2)	3 (7)	38 (90)
2007 (ACT implementation) (N = 63)	0 (0)	50 (79)	13 (21)
2008–2009 (ITNs scale up) (N = 16)	15 (94)	0 (0)	1 (6)
2010–2011 and 2013–2014 (malaria upsurges) (N = 28)	2 (7)	6 (21)	20 (71)
Symptomatic (clinical cases)			
2005 (before ACT) (N = 51)	5 (10)	18 (35)	28 (55)
2007 (ACT implementation) (N = 74)	0 (0)	8 (11)	66 (89)
2008–2009 (ITNs scale up) (N = 67)	57 (85)	0 (0)	10 (15)
2010–2011 and 2013–2014 (malaria upsurges) (N = 145)	26 (18)	2 (1)	117 (81)
All			
2005 (before ACT) (N = 93)	6 (6)	21 (23)	66 (71)
2007 (ACT implementation) (N = 137)	0 (0)	58 (48)	79 (52)
2008–2009 (ITNs scale up) (N = 83)	72 (87)	0 (0)	11 (13)
2010–2011 and 2013–2014 (malaria upsurges) (N = 173)	28 (16)	8 (5)	137 (79)

For clinical malaria cases, mixed infections were more frequent in 2007 and during malaria upsurges than in 2005, but were least frequent during the period of ITN scale-up (2008–2009). The frequency of mutant and mixed infections showed a similar trend in symptomatic and asymptomatic individuals, except for 2007, during ACT implementation, where mutant and mixed isolates were higher in asymptomatic carriers and in malaria cases, respectively (Table 3).

### *Pfcrt* codon 72–76 haplotype prevalence

**Fig. 3:**
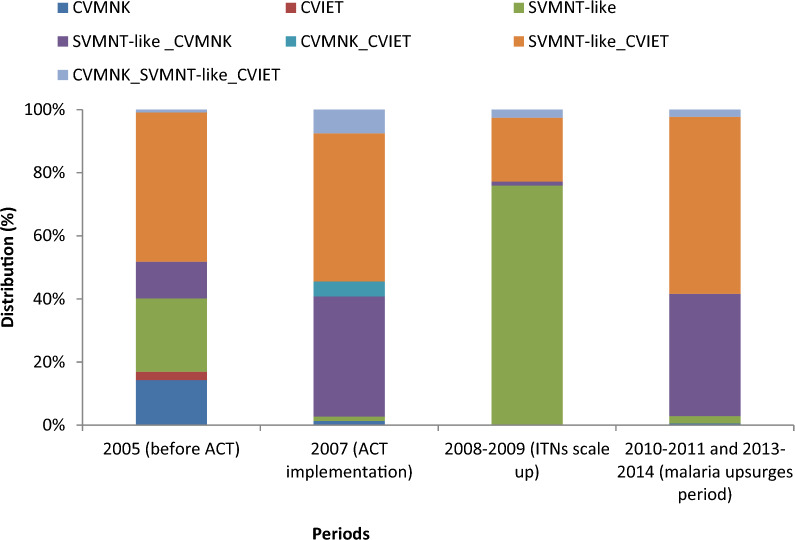
Distribution of *Pfcrt* haplotype in Dielmo *Plasmodium falciparum* isolates by period (511 genotypes)

Most samples (94.8%, 511/539) were successfully genotyped for the *Pfcrt* gene haplotype. In 2005, the wild type CVMNK was found in almost 30% of asymptomatic carriers but in only 6% of clinical malaria cases. The wild type was hardly detected over the remaining years (Table 4). The SVMNT-like CVMNK and the SVMNT-like_CVIET mixed haplotypes were the most frequent among asymptomatic carriers (The SVMNT haplotype has been observed mainly in Asia and rarely in Angola, Tanzania and Cameroon [18, 31–33]; therefore it is called SVMNT-like by precaution). Among clinical cases, the haplotype SVMNT-like_CVIET was most common, while the SVMNT-like was found in 83% of infections in 2008–2009 (Table 4). There was no obvious temporal trend although, when considering both asymptomatic carriers and clinical cases, SVMNT-like_CVIET was consistently present across the study period at relatively high frequency, except in 2008–2009 (Fig. 3, Table 4). The frequency of the mixed haplotypes SVMNT-like_CVMNK and SVMNT-like_CVIET was particularly low in 2008–2009, after implementation of ACT and during the scale-up of ITNs. Their frequency increased significantly (P < 0.001) during the malaria upsurges, in 2010–2011 and 2013–2014, reaching higher levels than those observed in 2007, the year of ACT implementation (Table 4).

**Table 4:** Prevalence (%) of the *Pfcrt* gene mutations by period in asymptomatic carriers and clinical cases

	CVMNK	CVIET	SVMNT-like	SVMNT-like _CVMNK	CVMNK_CVIET	SVMNT-like_CVIET	CVMNK_SVMNT-like _CVIET
Asymptomatic carriers							
2005 (before ACT) (N = 42)	12 (29)	1 (2)	7 (17)	13 (31)	0 (0)	9 (21)	0 (0)
2007 (ACT implementation) (N = 67)	1 (1)	0 (0)	0 (0)	52 (78)	7 (10)	1 (1)	6 (9)
2008–2009 (ITNs implementation) (N = 16)	0 (0)	0 (0)	8 (50)	0 (0)	0 (0)	6 (38)	2 (13)
2010–2011 and 2013–2014 (malaria upsurges) (N = 28)	1 (4)	0 (0)	0 (0)	17 (61)	0 (0)	10 (36)	0 (0)
Symptomatic (clinical cases)							
2005 (before ACT) (N = 70)	4 (6)	2 (3)	19 (27)	0 (0)	0 (0)	44 (63)	1 (1)
2007 (ACT implementation) (N = 80)	1 (1)	0 (0)	2 (3)	4 (5)	0 (0)	68 (85)	5 (6)
2008–2009 (ITNs implementation) (N = 63)	0 (0)	0 (0)	52 (83)	1 (2)	0 (0)	10 (16)	0 (0)
2010–2011 and 2013–2014 (malaria upsurges) (N = 145)	0 (0)	0 (0)	4 (3)	50 (34)	0 (0)	87 (60)	4 (3)
All							
2005 (before ACT) (N = 112)	16 (14)	3 (3)	26 (23)	13 (12)	0 (0)	53 (47)	1 (1)
2007 (ACT implementation) (N = 147)	2 (1)	0 (0)	2 (1)	56 (38)	7 (5)	69 (47)	11 (7)
2008–2009 (ITNs implementation) (N = 79)	0 (0)	0 (0)	60 (76)	1 (1)	0 (0)	16 (20)	2 (3)
2010–2011 and 2013–2014 (malaria upsurges period) (N = 173)	1(1)	0(0)	4(2)	67(39)	0(0)	97(56)	4(2)

## Discussion

Determining the prevalence of specific parasite mutations or haplotype driving anti-malarial drug resistance can inform on the effect of drug interventions and provide signal on how malaria parasite populations are evolving under drug pressure. ACT is currently the only recommended treatments for uncomplicated malaria and any resistance to artemisinin and its partner drugs will constitute a serious threat to malaria control and elimination. This study describes the evolution of molecular markers associated with anti-malarial drugs resistance in a Senegalese village at a time of substantial changes of both the malaria burden and various malaria control interventions.

In Senegal, between 2005 and 2014, the first-line treatment changed from a combination of SP and AQ to ACT, while ITNs and other interventions were scaled up, including seasonal malaria chemoprevention (SMC). This resulted in a substantial decrease of the malaria burden [5, 6, 34], to the point that the NMCP is currently planning to achieve malaria elimination by 2030. Such changes occurred also in Dielmo, a small village in the Fatick region that has been extensively studied to characterize the dynamic of malaria transmission [23, 27]. Indeed, malaria prevalence in Dielmo decreased substantially over the years, from 72%, in 1990, at the beginning of the project to less than 1% in 2012 [23]. Therefore, the intensive follow-up implemented in Dielmo provides the opportunity to monitor the evolution of anti-malarial drug resistance markers during a period of substantial malaria epidemiological changes. The prevalence of several molecular markers associated to drug resistance [35, 36] changed substantially during the study period, dropping particularly after the scale-up of ITNs and rebounding during the malaria upsurges, in 2010–2011 and 2013–2014, illustrating how multiple selection pressures due to various control interventions can shape the parasite population, as well as the quick regain in fitness of establish drug resistant parasite once interventions are perturbed [37].

The prevalence of drug resistance molecular markers was roughly similar between asymptomatic carriers and clinical cases, except in 2007, at the time of ACT implementation. When considering these two groups together, very few infections carried the wild type alleles, except at codon 184 of *Pfmdr1,* which was most frequent in 2008–2009, following the scale-up of ITNs. Nevertheless, the *Pfmdr1* N86 wild type was only detected in 2005, before the implementation of ACT, while most infections were mutant or mixed during the following years. Replacement of *Pfmdr1* N86 wild type isolates with 86Y mutant following the implementation of ACT is indicative of directional selection by artemisinin partner drugs such as AQ and LUM. It has been shown previously that AQ pressure selects for *Pfmdr1* 86Y mutant parasites while LUM selects for the N86 wild type [14, 35, 38]. The *Pfcrt* mutations associated with chloroquine resistance followed a similar pattern.

Before and just after the implementation of ACT, there were significantly high frequencies of the *Pfmdr1* and *Pfcrt* mutant and mixed type infections. This is probably due to the high transmission during that period, large number of clinical cases, drug pressure and selection of resistance parasites from earlier implementation of CQ monotherapy (between 1995 and 2003), and the use of the SP and AQ combination from the end of 2003 to May 2006. AQ resistance is mainly associated with the *Pfmdr1* 86Y mutation, and partly with mutations in the *Pfcrt* SVMNT haplotype [14, 18, 39, 40], while CQ resistance is mainly associated with 76 T mutant codon at *Pfcrt* gene [15]. These results differ from those obtained in other sites in Senegal (Dakar and Thiès) where the prevalence of the *Pfmdr1* 86Y allele was high between 2000 and 2003 but decreased afterwards following the withdrawal of CQ [19, 41]. Dakar and Thiès are large urban centres, where transmission is much lower and adherence to recommended treatment may have allowed for the differences observed. In Malawi, discontinuing CQ use resulted in a decrease of the prevalence of drug resistance markers and restored CQ efficacy [42]. However, in Dielmo this phenomenon was not observed. CQ resistance markers remained high, with the rapid dissemination of the *Pfcrt* resistance allele, which increased from 8% in 1993 to 46% following the reintroduction of CQ in 1995 [43]. Though other reports show decreasing CQ resistance (both in vitro and prevalence of molecular markers) in other parts of the country, the continuous presence of the *Pfcrt* resistance alleles ten years after withdrawing CQ as first-line treatment in Dielmo is not in favour of the reintroduction of this treatment [44, 45]. It should be noted however that some *Pfcrt* resistance alleles especially those in SVMNT haplotype largely uncommon in West Africa are known to be associated with AQ resistance [18, 33, 46, 47]. The SVMNT haplotype has been observed mainly in Asia and sometimes in Tanzania, Angola and Cameroon [18, 31–33]. There is a need to sequencing samples from West Africa to inform about the presence of this haplotype since it has an important implication on AQ resistance knowing that AQ is used in many countries as the artemisinin-based combination.

Following the large-scale implementation of ITNs, just before the malaria upsurges, the malaria burden was at its lowest and the wild type *Pfmdr1* Y184 allele was the most prevalent, indicating a relatively unaffected parasite population at this locus. During the same period, only one type of allele was observed for *Pfmdr1* 86 although it was the *Pfmdr1* 86F-like [18, 48]. This mutation is rare and was not found yet in field samples in West Africa; it is called 86F-like in this study since sequencing to confirm this result was not possible because of the unavailability of the samples (mostly from TBS) and resources. The contribution of the *Pfmdr1* 86F allele towards AQ resistance is still unknown [18]. The high frequency of SVMNT-like haplotype and the presence of the *Pfmdr1* 86 F-like mutant during the period of the large-scale implementation of ITNs underline the continuous presence of mutations associated mainly with AQ resistance, while the CVIET haplotype mainly associated with CQ resistance was not found [14, 46]. Conversely, in Dakar and in Thiès, the prevalence of the mutant allele *Pfmdr1* 86Y gene decreased between 2003 and 2009 [19, 41] while in Dielmo it disappeared after 2007 and was found only in one sample during the malaria upsurges.

During the two malaria upsurges, parasites with the *Pfmdr1* 184 wild type were almost completely replaced with the mixed type, and two mixed types of *Pfmdr1* 86 became predominant. The *Pfcrt* gene was marked by the replacement of the mutant SVMNT-like haplotype by two mixed types. The presence of few mutant parasites and mostly mixed parasites within the *Pfmdr1* gene during this period could be associated with artemisinin drug pressure and/or AQ use, as no mutant *Pfmdr1* 184 was found during 2008–2009. It has been suggested that *Pfmdr1* gene amplification may contribute to decreased sensitivity to artemisinin [49, 50]. In this study however, only SNPs were genotyped, which are more relevant to response to artemisinin partner drugs. In Thiès (Senegal), between 2008 and 2011, and in Dakar, between 2010 and 2011, the prevalence of the *Pfmdr1* mutant type F184 was found to be high [20, 51, 52]. Isolates from Thiès with this mutant allele also had lower sensitivity to AQ, artemisinin and CQ [20]. SP and AQ were used in Dielmo between 2003 and 2005 before replacement with ASAQ combination during the following years. Given the high adherence to treatment in Dielmo (~ 85% for patients on anti-malarials), significant selection pressure would have been maintained on the parasite population [43].

Polymorphisms observed in *Pfmdr1* and *Pfcrt* genes before and during ACT implementation were most likely associated with AQ and CQ use and selection while those observed during the malaria upsurges were associated with continuous pressure in the implementation of ASAQ artemisinin-based combination. With verbal reports of unusual longer recovery times of patients on ASAQ treatment during the malaria upsurge periods (Mamadou Senghor, pers. commun.), monitoring these variants remain essential for malaria elimination. *Ex-vivo* and/or in vitro tests would be needed for additional evidence of association of these mutations with the treatment response against specific anti-malarial drugs.

The malaria upsurges in Dielmo ended after the mass distribution campaigns of ITNs in July 2011 and August 2014 for the first and second upsurges, respectively. The village is currently in the pre-elimination phase with current malaria prevalence less than 1% and no local malaria case observed between 2018 and 2021. However, anti-malarial resistance associated markers should be periodically monitored to identify reintroduction from surrounding villages.

Although, this study is limited in its inability to perform sequencing for verification, it is indicative of the dynamics of drug resistance markers following various interventions and changes in disease endemicity. Conversely, prospective studies with better quality specimens (other than TBS) will continue to inform on the current trends to better inform strategies being implemented to maintain the low incidence towards elimination.

## Conclusion

The prevalence of drug resistance markers was high in Dielmo, particularly during the malaria upsurges. Monitoring drug resistance markers should be conducted periodically to detect threats of emergence or resurgence that could compromised the efficacy of anti-malarial drugs.

### Supplementary Information

**Additional file 1: Table S1.** Control strains per marker.

**Additional file 2: Table S2.** Nucleotide sequences and sizes of primers from *Pfcrt* and *Pfmdr1* used to detect point mutations in *Plasmodium falciparum* genes associated with antimalarial drugs resistance.

## Data Availability

Data can be made available upon request to the corresponding author.
