# Supplementary material for: Rebound of multiple infections and prevalence of anti-malarial resistance associated markers following malaria upsurges in Dielmo village, Senegal, West Africa
Source: Malar J. 2023 Sep 5;22:257. doi: 10.1186/s12936-023-04694-0 (PMC10478411; doi:10.1186/s12936-023-04694-0)
Supplement: Supplementary file 1 — Additional file 1: Table S1. Control strains per marker. [file 12936_2023_4694_MOESM1_ESM.docx]

Supplementary Table 1: Control strains per marker

| Markers | Wild controls | Mutant controls |
| --- | --- | --- |
| *Pfcrt* | 3D7 | W2; Dd2 and TM90 |
| *Pfmdr1* 86 | 3D7 and HB3 | Dd2 and K1 |
| *Pfmdr1* 184 | 3D7 and Dd2 | HB3 and TM90 |
