# Supplementary material for: Rebound of multiple infections and prevalence of anti-malarial resistance associated markers following malaria upsurges in Dielmo village, Senegal, West Africa
Source: Malar J. 2023 Sep 5;22:257. doi: 10.1186/s12936-023-04694-0 (PMC10478411; doi:10.1186/s12936-023-04694-0)
Supplement: Supplementary file 2 — Additional file 2: Table S2. Nucleotide sequences and sizes of primers from Pfcrt and Pfmdr1 used to detect point mutations in Plasmodium falciparum genes associated with antimalarial drugs resistance. [file 12936_2023_4694_MOESM2_ESM.docx]

Supplementary Table 2: Nucleotide sequences and sizes of primers from *Pfcrt* and *Pfmdr1* used to detect point mutations in *Plasmodium falciparum* genes associated with antimalarial drugs resistance.

| Primer Name | Primer sequences (5'-3') | Targeted gene | No. Bases |
| --- | --- | --- | --- |
| pfcrt C72/M74/N75/K76 Fwd | GTA AAA CGA CGG CCA GTT TCT TGT CTT GGT AAA TGT GCT CA | *Pfcrt* | 41 |
| pfcrt C72/M74/N75/K76 Rev | CAG GAA ACA GCT ATG ACC GGA TGT TAC AAA ACT ATA GTT ACC AAT | *Pfcrt* | 45 |
| pfMDR N86 Fwd | TTA TTA TTT ATA TCA TTT GTA TGT GCT GTA TTA TCA GG | *PfMDR* N86 | 38 |
| pfMDR N86 Rev | CAG GAA ACA GCT ATG ACA TCA TTG ATA ATA TAA ATT GTA CTA AAC CTA TA | *PfMDR* N86 | 50 |
| pfMDR Y184 Fwd | AGT TCA GGA ATT GGT ACG AAA TTT ATA ACA | *PfMDR* Y 184 | 30 |
| pfMDR Y184 Rev | ACG CAA GTA ATA CAT AAA GTC AAA CG | *PfMDR* Y 184 | 26 |
